# Sex as a Critical Variable in Basic and Pre-Clinical Studies of Fibrodysplasia Ossificans Progressiva

**DOI:** 10.3390/biom14020177

**Published:** 2024-02-01

**Authors:** Lorraine N. Burdick, Amanda H. DelVichio, L. Russell Hanson, Brenden B. Griffith, Keith R. Bouchard, Jeffrey W. Hunter, David J. Goldhamer

**Affiliations:** 1 Department of Molecular & Cell Biology, University of Connecticut Stem Cell Institute, University of Connecticut, Storrs, CT 06269, USA; lorraine.apuzzo@uconn.edu (L.N.B.); amanda.delvichio@uconn.edu (A.H.D.); leslie.hanson_iv@uconn.edu (L.R.H.); brenden.griffith@uconn.edu (B.B.G.); 2 Alexion Pharmaceuticals Inc., 100 College Street, New Haven, CT 06510, USA; keith.bouchard@alexion.com (K.R.B.); jeffrey.hunter@alexion.com (J.W.H.)

**Keywords:** fibrodysplasia ossificans progressiva, FOP, ACVR1, ALK2, antibody therapeutics, heterotopic ossification, activin A, fibro-adipogenic progenitors, FAPs, BMP2, BMP6, BMP signaling, sex difference, sex dimorphism, gender difference, endochondral ossification

## Abstract

Heterotopic ossification (HO) is most dramatically manifested in the rare and severely debilitating disease, fibrodysplasia ossificans progressiva (FOP), in which heterotopic bone progressively accumulates in skeletal muscles and associated soft tissues. The great majority of FOP cases are caused by a single amino acid substitution in the type 1 bone morphogenetic protein (BMP) receptor ACVR1, a mutation that imparts responsiveness to activin A. Although it is well-established that biological sex is a critical variable in a range of physiological and disease processes, the impact of sex on HO in animal models of FOP has not been explored. We show that female FOP mice exhibit both significantly greater and more variable HO responses after muscle injury. Additionally, the incidence of spontaneous HO was significantly greater in female mice. This sex dimorphism is not dependent on gonadally derived sex hormones, and reciprocal cell transplantations indicate that apparent differences in osteogenic activity are intrinsic to the sex of the transplanted cells. By circumventing the absolute requirement for activin A using an agonist of mutant ACVR1, we show that the female-specific response to muscle injury or BMP2 implantation is dependent on activin A. These data identify sex as a critical variable in basic and pre-clinical studies of FOP.

## 1. Introduction

Many aspects of human biology under both normal physiological conditions and disease states exhibit sex dimorphism, yet sex as a biological variable often has not been considered in health-related research. The sex of experimental animals used in basic and pre-clinical studies is frequently restricted to a single sex or is not noted [1]. Inadequate consideration of sex-based differences in pharmacokinetics, pharmacodynamics, and drug interactions can lead to adverse effects, including fatalities, in response to treatments or interventions [2,3,4]. The growing documentation of biological processes and responses to treatment modalities that exhibit sex-specific differences has prompted the National Institutes of Health to announce the formal expectation that sex be considered a biological variable in vertebrate and human studies [5].

Sex differences have been observed in the incidence and severity of heterotopic ossification (HO)—a condition defined by the development of bone at extraskeletal soft tissue sites—of both non-genetic (or acquired) and genetic etiologies. Specifically, numerous clinical studies of non-genetic HO have reported male sex as a risk factor. Males exhibit both a higher incidence of HO and a more robust HO response after procedures such as cervical disk replacement/arthroplasty [6,7,8], hip arthroplasty [9,10], femoral intramedullary rodding [11], and injuries that include traumatic brain injury [12] and burns [13]. HO was observed exclusively in males following rhBMP7 treatment of long bone non-unions [14]. Finally, male individuals with Albright Hereditary Osteodystrophy (AHO) exhibit much more extensive subcutaneous HO than females [15].

Sex dimorphism in HO formation has also been documented in pre-clinical mouse studies. The extent of subcutaneous HO in a mouse model of AHO is greater in males [16]. Males were more susceptible to HO and showed a greater average HO volume in a burn–tenotomy model [17]. In addition, cultured mesenchymal stem cells derived from male mice after burn injury displayed greater osteogenic potential than female-derived cells [17]. In an independent study, cultured male muscle-derived stem cells showed greater osteogenic activity than female cells in response to BMP4 [18]. In contrast to these examples, mineralized HO volume was greater in females following intramuscular implantation of BMP2 [19].

HO is most dramatically manifested in the severely debilitating disorder, fibrodysplasia ossificans progressiva (FOP; OMIM 135100), which is characterized by progressive HO of skeletal muscles, tendons, and ligaments, typically beginning in early childhood. The great majority of FOP cases are caused by an arginine to histidine substitution at position 206 (R206H) of the BMP type 1 receptor, ACVR1 (also known as ALK2) [20,21]. This amino acid change in the intracellular GS domain alters receptor responsiveness to activin A, resulting in the activation of BMP signaling and the development of heterotopic bone [22,23]. Although sexual dimorphism has not been reported in disease prevalence or severity [24,25,26], a preliminary retrospective survey in ACVR1(R206H) FOP mouse models suggested a possible female sex bias in the volume of injury-induced HO. In these mice, the expression of *Acvr1^R206H^* was targeted to fibro-adipogenic progenitors (FAPs), multipotent, tissue-resident, mesenchymal cells [27,28] that are a major source of skeletal progenitors in both FOP and acquired HO models [29,30,31,32,33]. Here, using a range of experimental conditions, we show that female FOP mice produce a significantly greater volume of HO than male mice and are more susceptible to spontaneous HO (HO that forms without experimentally imposed injury or other known triggers). Further, the female sex bias in injury-induced HO is dependent on activin A. The results emphasize the importance of considering sex as a key variable in basic and pre-clinical studies of FOP disease pathogenesis and drug efficacy. 

## 2. Materials and Methods

### 2.1. Experimental Mouse Models and Genotyping

Experimental mice utilizing the Tie2-Cre driver were generated by crossing *Acvr1^tnR206H/+^* female breeders [30] to male Tie2-Cre transgenic mice [34], a gift of Dr. Tom Sato (UT Southwestern). For mice used in flow cytometry analysis or fluorescence-activated cell sorting (FACS), we incorporated the lineage reporters, *Pdgfra^H2B-eGFP^* (*Pdgfra^H2B-GFP^*) [35] (JAX #007669) or *R26^NG^* [36], respectively. For in vitro cell proliferation assays, we incorporated the luciferase reporter, *R26^luc^* [37] (JAX #005125). SCID Hairless Outbred mice (SHO*-Prkdc^scid^Hr^hr^*: hereafter, SCID) (Charles River: strain code 474) were used as recipients for male–female reciprocal transplantations. Adult mice used in this study were 8- to 12- weeks-of-age, except for the study of HO in older adults, which were 12- to 14- months-of-age. Experimental mice were on a predominantly FVB background. Mice were genotyped using PCR and reporter fluorescence, as previously described [30]. 

Experimental *Acvr1^FLEx(R206H)/+^*;CAG-Cre^ERT2^ mice were generated by crossing heterozygous *Acvr1^FLEx(R206H)^* females to heterozygous B6.Cg-Tg(CAG-cre/Esr1*)5Amc/J (JAX #04682) [38] males to generate mice that were heterozygous at both loci, as described previously [39]. Experimental mice were on a hybrid 129Sv/C57BL/6 background.

### 2.2. Tamoxifen Administration

To induce recombination at the *Acvr1^FLEx(R206H)^* locus, 75 mg/kg tamoxifen (Sigma-Aldrich, #T5648; St. Louis, MO, USA) prepared as a 20 mg/mL stock in corn oil (Sigma-Aldrich, #C8267) was administered via intraperitoneal injection to experimental mice for 5 consecutive days. A 5- to 7-day washout period was incorporated prior to muscle injury [39].

### 2.3. Muscle Injury

Hindlimb muscles of *Acvr1^tnR206H/+^*; *R26^NG/+^*;Tie2-Cre mice were injured either by pinch injury or cardiotoxin injection. For pinch injury, a force of approximately 3800–4000× *g* was applied to the mid-belly of the gastrocnemius (GA) muscle using a Randall Selitto Paw Pressure Test Apparatus (IITC Life Science, Woodland Hills, CA, USA) while the mice were under isoflurane anesthesia, as described [30]. A separate cohort of *Acvr1^tnR206H/+^*; *R26^NG/+^*;Tie2-Cre mice was injured by injection of 50 μL of 10 μM cardiotoxin (Latoxan, #L8102-1MG; Portes-lès-Valence, France) in sterile 1X Dulbecco’s phosphate-buffered saline (PBS; Gibco; Grand Island, NY, USA) into the tibialis anterior (TA) muscle. To induce muscle injury in *Acvr1^FLEx(R206H)/+^*;CAG-Cre^ERT2^ mice, 100 μL of 10 μM cardiotoxin (Sigma-Aldrich, #C9759) was injected into the GA muscle.

### 2.4. Ligand and Antibody Injections

Mice subjected to BMP treatment were injected with a 100 μL suspension consisting of 2.5 µg rhBMP2 (Pfizer, New York City, NY, USA) or rhBMP6 (Keros Therapeutics; Lexington, MA, USA) suspended in sterile 1X PBS and 1% alum adjuvant solution (Invivogen, Alhydrogel^®^ adjuvant 2%; San Diego, CA, USA). BMP adsorption to the adjuvant carrier was facilitated by gentle shaking at room temperature for ~15 min prior to use. Injections were made into the GA muscle with or without 10 mg/kg anti-activin A monoclonal antibody (ActA-mAb; Acceleron Pharma; Cambridge, MA, USA) delivered subcutaneously at the time of BMP injection. Mice subjected to treatment with the anti-ACVR1 antibody JAB0505 were given a single intraperitoneal dose of JAB0505 (10 mg/kg) on the day of injury, as described previously [39]. ActA-mAb (10 mg/kg) was administered via subcutaneous injection on the day of injury and at 14 days post-injury. ActA-mAb [30] and JAB0505 [39] were described previously.

### 2.5. µCT and HO Quantification

µCT images were acquired using either an IVIS Spectrum-CT (Perkin Elmer; Hopkinton, MA, USA) or a Quantum FX μCT Cabinet X-ray System (Perkin Elmer) as previously described [39]. Here, 3D Slicer V4.0 software (www.slicer.org) was used to segment and quantify heterotopic bone volume (mm^3^). 

### 2.6. Cell Isolation

A single-cell suspension was obtained using the tissue dissociation methods described previously [30,31,32,40], with slight modifications. The GA was minced with scissors in a 35 mm dish containing ~100 μL dissociation media consisting of Dulbecco’s Modified Eagle Medium (DMEM; Thermo Fisher; Waltham, MA, USA), 600–700 U/mL Collagenase Type II (Worthington Biochemical; Lakewood, NJ, USA), and 0.5 U/mL Dispase (Thermo Fisher) for 3 min and placed on ice. Minced tissue was then incubated in 10 mL dissociation media at 37 °C with gentle agitation for 1 h. Enzymatic digestion was quenched via the addition of growth media (DMEM containing 20% HyClone characterized fetal bovine serum (FBS; GE Healthcare, Lot #A00168; Chicago, IL, USA) followed by serial filtration of the cell suspension through 100 µm and 70 µm cell strainers (Corning Life Sciences; Tewksbury, MA, USA). 

### 2.7. Flow Cytometry Analysis

The GA muscle of *Acvr1^tnR206H/+^*; *Pdgfra^H2B-GFP^*;Tie2-Cre mice was collected and weighed before tissue dissociation (above). We alternated sex, genotype, and condition during collection to account for potential differences in viability from the length of time from harvest to analysis. The single-cell suspension was centrifuged at 500× *g* for 5 min and resuspended in 10% FBS in sterile 1X PBS for antibody staining. Cell suspensions were incubated with the following fluorescently conjugated antibodies: anti-CD31-BV711 (BD Biosciences; 1:800, #740680; San Jose, CA, USA), anti-CD45-BV711 (BD Biosciences; 1:500, #563709), and SCA-1-V450 (1:400, BD Biosciences, #560653). Cells were incubated for 30 min on ice, centrifuged at 500× *g* for 5 min, and resuspended in 500 μL of 2% FBS in 1X DPBS following one wash with 1X DPBS. Samples were filtered through 35 µm cell strainers (Corning Life Sciences), and 50 µg/mL 7-AAD (BioLegend) was added to a final concentration of 0.50 µg/mL immediately prior to sorting to distinguish between live and dead cells. To obtain an absolute cell count, 50 µL of Precision Count Beads™ (984 beads/μL, Biolegend, #424902; San Diego, CA, USA) was added to 500 µL of each sample. Bead concentration was verified using a hemocytometer. Tubes were gently vortexed immediately prior to analysis. All samples were analyzed on a BD LSR Fortessa X-20 (BD Biosciences, Franklin Lakes, NJ, USA) equipped with 355, 405, 488, 561, and 633 nm lasers. Single color and fluorescence-minus-one (FMO) controls were used for fluorescence compensation and gating, respectively. Data were analyzed using FlowJo software (v10.6.1).

### 2.8. Fluorescence-Activated Cell Sorting

The total hindlimb muscle of uninjured *Acvr1^tnR206H/+^;R26^NG/+^*;Tie2-Cre or *Acvr1^tnR206H/+^*; *R26^luc/+^*;Tie2-Cre mice was collected and dissociated as described above. The crude muscle suspension was plated on plastic tissue culture dishes and incubated at 37 °C in a humidified atmosphere with 5% CO_2_. The growth media consisted of 20% premium FBS (R&D Systems, Lot #C19032; Minneapolis, MN, USA) in DMEM supplemented with 50 U/mL penicillin and 50 μg/mL streptomycin (Pen/Strep; Gibco), as previously described [30,31]. After ~12–16 h of incubation in growth media, non-adherent cells were washed from the plate 2–3X with warm DPBS, and fresh growth media was added to each plate. Cells were subjected to media changes every other day and were carefully monitored to avoid surpassing 70% confluency. Once cells had reached 70% confluency, approximately 5 days post-harvest, plates were washed 1X with DPBS and cells were enzymatically detached with Accumax (Innovative Cell Technologies; San Diego, CA, USA) to obtain a single-cell suspension. Each sample was prepared for FACS using the methods described for flow cytometry analysis, except that PDGFRα-APC antibody (1:100, eBioscience, #17-1401-81; San Diego, CA, USA) was used in place of *Pdgfra^H2B-GFP^*. Live, mononuclear, CD31^-^/CD45^-^/tdTomato^-^/PDGFRα^+^/SCA-1^+^ FAPs were collected in sterile 2% FBS in DPBS using a FACS Aria II (BD Biosciences) equipped with 355, 405, 488, 561, and 633 nm lasers and plated in growth media immediately after collection. Single color and FMO controls were used for fluorescence compensation and gating, respectively.

### 2.9. Reciprocal Transplantation

After the initial growth period in culture and FACS isolation, *Acvr1^R206H^*-expressing FAPs (R206H-FAPs) were cultured for an additional 2–3 days in growth media until cells reached ~70% confluency. The maximum time cells were in culture was approximately 12–14 days after cell isolation. Immediately prior to transplantation, SCID mice were pinch-injured, as described above. After injury, 7.5 × 10^5^ male or female R206H-FAPs suspended in 50 μL sterile 1X DPBS were transplanted into the GA muscle of the right or left hindlimb, respectively, of male and female SCID hosts. Mice were scanned using µCT at day 15 post-injury. 

### 2.10. Castration Surgery

8- to 12-week-old male mice were subjected to continuous 2.5% isoflurane sedation at 0.8 L/min in a dorsal recumbent position during the castration procedure [41]. Meloxicam analgesic (5 mg/kg) was administered subcutaneously immediately after sedation and 24 h post-surgery. The surgical site was prepared using the standard surgical aseptic technique; the scrotum was depilated and washed 3X alternating betadine and 70% ethanol. Castration was achieved via the cauterization of the vas deferens and spermatic blood vessels in both testicles. Wound glue was used to close the skin. All mice were singly housed for the duration of the study and were allowed to recover for 3 to 4 weeks prior to receiving experimental injury.

### 2.11. Ovariectomy

8- to 12-week-old female mice were ovariectomized following previously described guidelines [42]. The processes used for anesthesia, pre- and post-surgical analgesic, and preparation of the surgical site were the same as those used for the castrations. For each ovary, a 1–2 cm incision was made in the skin lateral to the midline, directly between the pubis and rib bones. A second 1–2 cm incision was made in the abdominal wall musculature, and using fine forceps, each ovary was gently removed from the abdomen. Ovaries were detached from the uterine horn via cauterization and discarded. The inner body wall was closed with non-absorbent sutures, and the skin was closed with wound glue. All mice were singly housed for the duration of the study and allowed to recover for 3 to 4 weeks prior to receiving experimental injury at approximately 12 to 16 weeks of age.

### 2.12. Luciferase Proliferation Assay

After FACS isolation, male and female FAPs were suspended in growth media and plated in triplicate at a density of 1000 cells/cm^2^ in a 96-well plate. Cells were allowed to recover for 24 h, and media was changed every other day for the course of the assay. At each time point collected, the growth medium was replaced with 300 μg/mL D-luciferin (Perkin Elmer; Waltham, MA, USA) in fresh, pre-warmed growth media and incubated for 2 min at 37 °C in 5% CO_2_. Luciferase absorbance values were determined using the SpectramaxPlus Plate Reader (Molecular Devices San Jose, CA, USA). Cell numbers were determined from a standard curve of luciferase absorbance relative to known cell concentrations. 

### 2.13. Statistics

Statistical analysis was performed using GraphPad Prism (GraphPad, La Jolla, CA, USA), except for the linear regression analysis, which was performed in RStudio (Posit Software, PBC). All numerical values are presented as mean values ± standard error of the mean. Where indicated, a one- or two-tailed unpaired *t*-test, Fisher’s exact test, two-way ANOVA, or one-way ANOVA with Tukey’s multiple-comparison test was used to determine significance. Differences were considered significant at *p* ≤ 0.05.

## 3. Results

### 3.1. Female FOP Mice Develop More Heterotopic Bone and Exhibit a More Variable Response to Muscle Injury Than Males 

**Figure 1:**
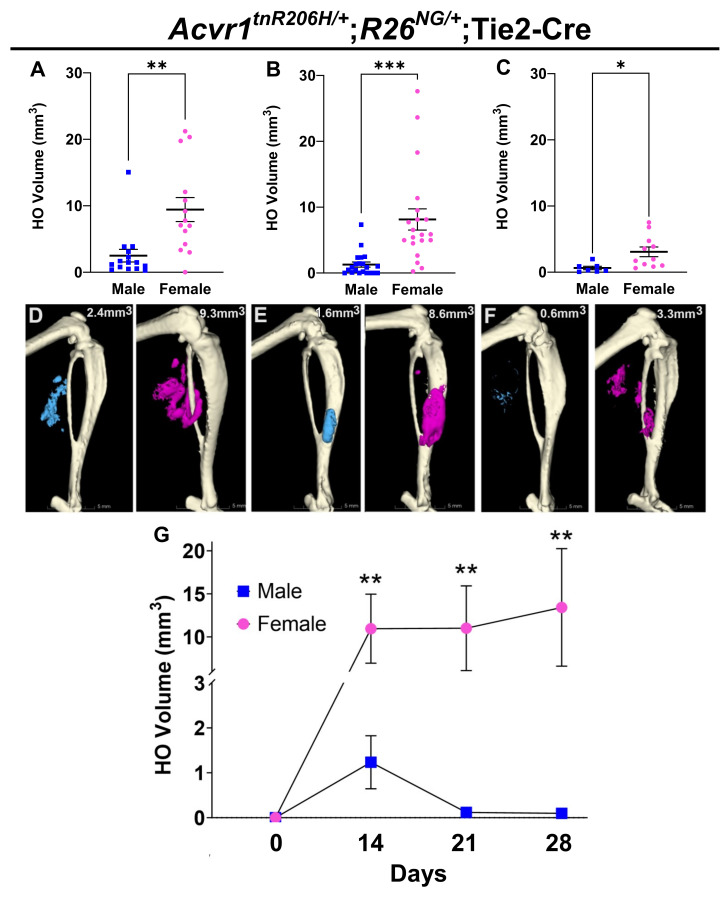
Female-biased heterotopic bone formation in FOP mice. (**A**) Quantification of heterotopic ossification (HO) volumes (mm^3^) 14 days after pinch-injury of the gastrocnemius (GA) muscle of 8- to 12-week-old FOP mice; n = 15 for males; n = 14 for females. (**B**) Average HO volumes 14 days after cardiotoxin-induced injury of 8- to 12-week-old FOP mice; n = 21 for males; n = 20 for females. (**C**) Average HO volume 21 days after pinch-injury of 12- to 14-month-old FOP mice; n = 7 for males; n = 11 for females. (**D**–**F**) Representative µCT images of HO in males (left panels) and females (right panels) from (**A**–**C**), respectively. HO was pseudocolored blue (males) and pink (females). (**G**) Time course of mean HO volumes from a separate cohort of pinch-injured *Acvr1^tnR206H/+^*;Tie2-Cre mice. For statistical analyses, a two-tailed unpaired *t*-test was used for data in (**A**–**C**), and two-way ANOVA was used for data in (**G**). * *p* < 0.05; ** *p* < 0.01; *** *p* < 0.001. Mean ± SEM.

To determine whether the extent of injury-induced HO in FOP mice was associated with sex, the GA muscle of 8- to 12-week-old mice was pinch-injured, and HO was quantified using μCT at day 14 post-injury, as previously described [30]. Mice of the genotype *Acvr1^tnR206H/+^*;Tie2-Cre were used, where *Acvr1^tnR206H^* is the Cre-dependent FOP allele [30] and Tie2-Cre [34] targets the recombination of *Acvr1^tnR206H^* to a subset of FAPs [30,32] and to endothelium and hematopoietic cells [30,32,34,43]; the latter two cell types do not directly contribute to HO in FOP mice [30,44]. Some mice also carried either the Cre-dependent GFP reporter, *R26^NG^* [36], or the luciferase reporter, *R26^luc^* [37], which was used for the enrichment of FAPs in transplantation studies or proliferation assays, respectively. As the magnitude of the HO response is positively correlated with the degree of injury in this model (unpublished observations), a standardized pinch force was applied using a Randall Selitto Paw Pressure Test Apparatus to reduce variability in HO volume at the endpoint. To assess technical variability, both GA muscles of each mouse were injured, and HO volumes were quantified at day 14 post-injury; variability in HO volumes between limbs was not statistically significant (Supplementary Figure S1A). For subsequent analyses using this model, each data point represents the average HO volume per mouse after bilateral muscle injury. Notably, the mean HO volume in females at the day 14 endpoint was approximately four-fold greater than that of males (Figure 1A,D,G; *p* < 0.01). In addition, female mice exhibited nearly two-fold greater variability in HO volumes than males (*p* < 0.05). Despite reduced injury-induced HO volumes in 12- to 14-month-old FOP mice, pinch injury of the GA muscle also resulted in significantly greater HO in female mice at 21 days post-injury (Figure 1C,F; *p* < 0.05).

We also conducted a longitudinal analysis of HO formation using a separate cohort of *Acvr1^tnR206H/+^*;Tie2-Cre mice to determine whether male–female differences in HO volumes persisted past the day 14 endpoint. This analysis addressed whether males and females differ regarding the time to peak volume of mineralized bone, or the degree of resorption of nascent bone, which is often observed in our FOP mouse models (unpublished observations). Male and female mice were pinch-injured as above, and HO volumes were quantified via μCT on days 0, 14, 21, and 28 post-injury. Female mice exhibited significantly greater mineralized bone volumes at all post-injury time points (Figure 1G; Supplementary Figure S2A–D). Further, whereas HO in male mice peaked at day 14 with substantial resorption of bone thereafter, female mice showed greater variability in the time to peak HO and in the extent of bone resorption (Figure 1G; Supplementary Figure S1D). In addition to demonstrating the persistence of male–female differences in HO volumes, this analysis highlights the importance of the choice of study endpoints and the stage-specific contributions of both HO growth and remodeling to final HO volumes.

In the age range of 8 to 12 weeks, male mice weighed on average 25% more than female mice (Supplementary Figure S1B). Although the muscle was injured using a standardized applied force, differences between males and females in muscle mass, although expected to be small in this narrow age range [45], and perhaps in the density or stiffness of intra- and inter-muscular connective tissues, might affect the degree and volume of muscle tissue damage and, consequently, could contribute to differences in HO volume at the endpoint. As muscle mass is proportional to body weight in male and female mice [46], we used linear regression analysis to determine possible correlations between sex, weight, and average HO volumes at day 14 post-injury. Using data from the cohort of mice in Figure 1A, linear regression analyses revealed a statistically significant relationship between sex and HO (*p* < 0.05) but not body weight and HO (*p* = 0.7; Supplementary Figure S1C). We also assessed the HO response following cardiotoxin-induced muscle injury under the assumption that, presumably small differences in muscle mass would not substantially influence the degree of chemically induced muscle damage. The TA muscle of 8- to 12-week-old male and female FOP mice was injected with cardiotoxin, and HO was quantified at 14 days post-injury. As with HO induced by pinch injury, a similar female-biased sex difference in HO volumes was observed (Figure 1B,E). Collectively, these data demonstrate a female bias in HO formation in FOP mice and further show that the response to injury is not restricted to a particular muscle, method of injury, or age of mice. 

### 3.2. Female Bias in Both Injury-Induced and Spontaneous HO in an Independently Derived FOP Mouse Model

**Figure 2:**
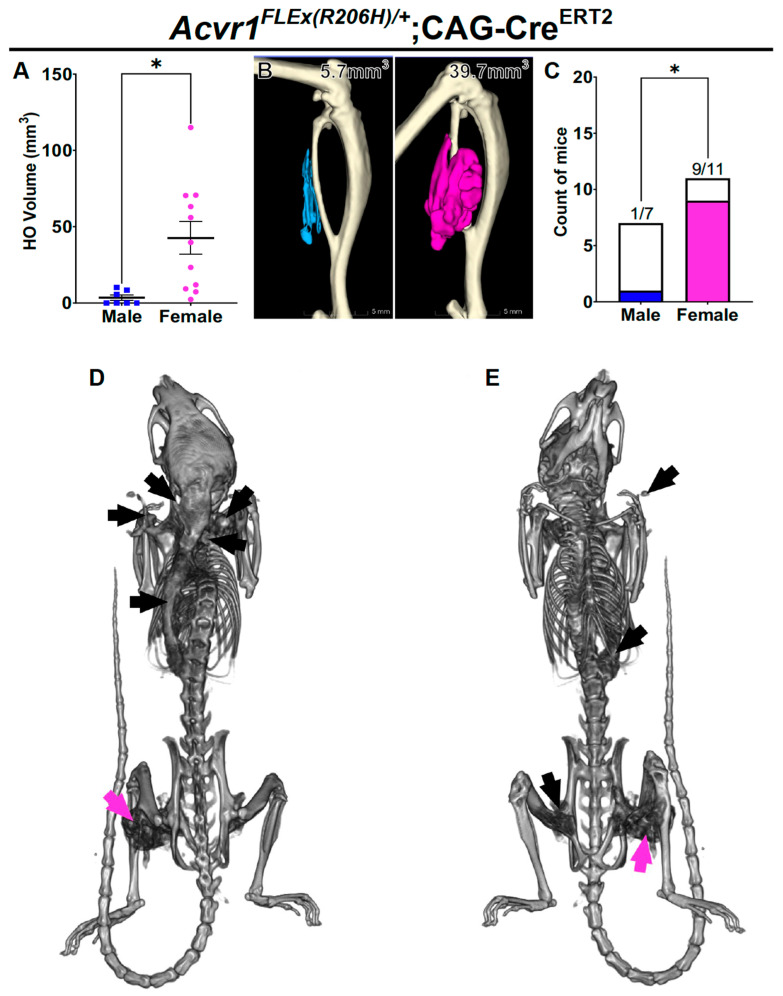
The female-biased HO phenotype is evident in an independent mouse model of injury-induced and spontaneous HO. (**A**) Quantification of HO 28 days after cardiotoxin-induced injury of the GA muscle of 8- to 12-week-old mice; n = 7 for males; n = 11 for females. (**B**) Representative µCT images of male (left, blue) and female (right, pink) HO. (**C**) Incidence of spontaneous HO in male (blue) and female (pink) FOP mice from (**A**) at 4 months post-injury. (**D**,**E**) Representative female from (**C**) showing locations of spontaneous HO (black arrows) and site of cardiotoxin-induced injury (pink arrows) from a dorsal (left) and ventral (right) view. For statistical analyses, either a two-tailed unpaired *t*-test (**A**) or Fisher’s exact test (**C**) was used. * *p* < 0.05. Mean ± SEM.

The unexpected female bias we observed in heterotopic bone formation raised the question of whether the sex difference was specific to either the *Acvr1^tnR206H^* allele or the use of the Tie2-Cre driver (or both), in which HO is predominantly derived from FAPs [30,33]. To address whether the result could be generalized to other FOP mouse models, we used the independently derived FOP mouse allele, *Acvr1^FLEx(R206H)^* [39], together with the ubiquitously expressed and tamoxifen-dependent CAG-Cre^ERT2^ transgenic driver [38]. Tamoxifen was administered to 4- to 8-week-old FOP mice (75 mg/kg for five consecutive days), and cardiotoxin was injected into the GA muscle after a 5- to 7-day washout period. A statistically greater HO volume in female mice was also observed in this model (*p* < 0.05), as assessed by μCT at 28 days post-injury (Figure 2A,B).

It is common for individuals with FOP to develop HO without a known trigger, which is often referred to as spontaneous HO [47]. To determine whether there was a sex bias in spontaneous HO in FOP mice, *Acvr1^FLEx(R206H)/+^*;CAG-Cre^ERT2^ mice were maintained after analysis of injury-induced HO to determine the frequency and extent of spontaneous HO, excluding the initial injury site from the analysis. Nine of the eleven female mice (82%) analyzed using µCT at 3 months post-injury (4- to 5-months-of-age) exhibited HO at sites anatomically distinct from the initial injury site (Figure 2C–E). In some cases, the body burden of spontaneous HO was extensive (Figure 2D,E). In striking contrast, only one of seven male mice (~14%) exhibited HO away from the initial injury site, and this HO was restricted to the base of the tail, a common site for biting among cage mates. Despite an atypical location of spontaneous HO [31], this male was included in the analysis because the cause of the HO could not be established with certainty. Nevertheless, the differential responses of females and males were significant (*p* < 0.05). These results highlight a substantial sex-specific difference in injury-induced HO and the incidence of spontaneous HO in this independent FOP mouse model.

### 3.3. The Female Sex Bias in HO Is Not Due to a Difference in the Abundance of FAPs or to a Greater Efficiency of Cre-Dependent Recombination of the Acvr1^tnR206H^ Allele

**Figure 3:**
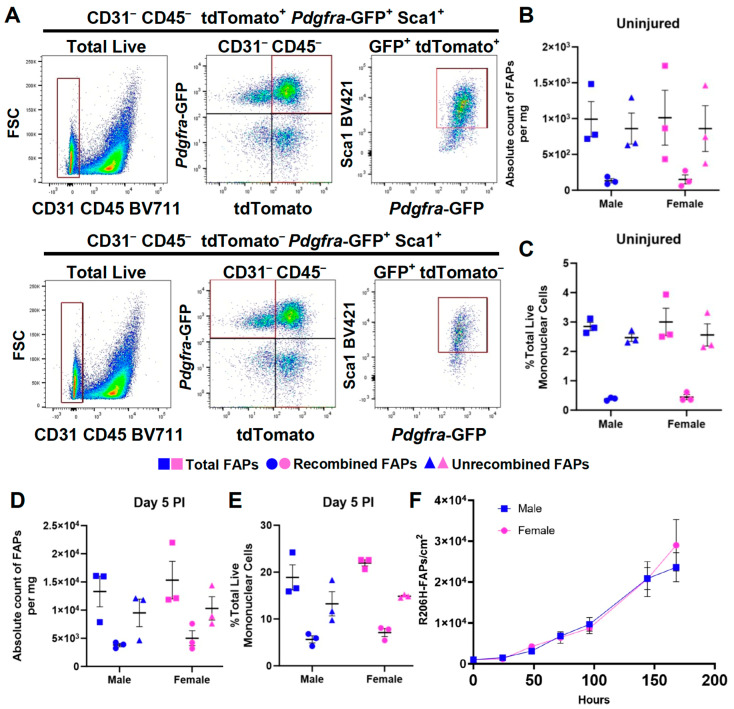
The absolute number and percentage of fibro-adipogenic progenitors (FAPs) is similar in male and female FOP mice. (**A**) Flow cytometry gating progression of total live mononuclear cells from *Acvr1^tnR206H/+^*;*Pdgfra^H2BGFP^^/+^*;Tie2-Cre mice. (**B**–**E**) Number of total, recombined (R206H-FAPs), and unrecombined FAPs in uninjured tissue (**B**,**C**) or 5 days after muscle injury (**D**,**E**) represented as an absolute number per mg of tissue (**B**,**D**) or as a percentage of total live mononuclear cells (**C**,**E**). PI, post-injury. n = 3 for males and females. (**F**) Growth curve of cultured R206H-FAPs isolated from *Acvr1^tnR206H/+^;R26^luc/+^*;Tie2-Cre mice by FACS. n = 3 technical replicates for each sex at each time point, performed in duplicate. A two-tailed unpaired *t*-test was used for statistical analyses. There were no statistically significant differences between sexes in (**B**–**F**). Mean ± SEM.

A priori, the greater HO response in females could be explained by a greater number or density of FAPs or a greater efficiency of Cre-mediated recombination, either of which would increase the representation of R206H-FAPs in muscle tissue. To test whether this can explain the female sex bias, mononuclear cells were isolated from total hindlimb muscles of FOP mice of the genotype *Acvr1^tnR206H/+^*;*Pdgfra^H2B-GFP^*;Tie2-Cre and analyzed using flow cytometry to quantify total, recombined, and unrecombined FAPs. In this experiment, the *Pdgfra^H2B-GFP^* allele [35] replaced the Cre-dependent GFP reporter, *R26^NG^* [36], allowing for the quantification of FAPs by the *Pdgfra*-driven expression of GFP after excluding CD45^+^ (hematopoietic) and CD31^+^ (endothelial) cells (Figure 3A). In hindlimb muscle tissue, the vast majority of PDGFRα^+^ cells are FAPs, and preliminary studies showed that *Pdgfra^H2B-GFP^* provides a cleaner fractionation of FAPs than the use of an antibody against endogenous PDGFRα. As single-cell RNA sequencing (scRNA-seq) showed that some cells of the peripheral nerve sheath in the hindlimb express *Pdgfra* (unpublished observations), SCA-1 was included in the flow cytometry analysis (Figure 3A) to distinguish FAPs from nerve sheath cells, which are SCA-1-negative. As Cre recombination of the *Acvr1^tnR206H^* allele excises the tdTomato-containing stop cassette [30], unrecombined FAPs and R206H-FAPs were identified by the presence and absence, respectively, of tdTomato-derived fluorescence [30,31].

The quantification of FAPs via flow cytometry revealed no sex differences in the number of total or R206H-FAPs in uninjured muscle, either as a percentage of total mononuclear cells or per milligram of muscle tissue (Figure 3B,C). To address whether FAPs accumulate to a greater extent in females after muscle injury, parallel experiments were conducted 5 days after pinch injury, just before the appearance of histologically identifiable cartilage in this model. No difference between males and females was observed for either recombined or unrecombined FAPs (Figure 3D,E). Analysis of R206H-FAP growth in cell culture also revealed no significant sex-specific differences, as assessed through 1 week in culture, when plates reached near-confluency (Figure 3F). These data indicate that sex differences in the extent of HO formation cannot be attributed to differential representation of FAPs in muscle tissue or to differences in growth dynamics at early, pre-skeletal post-injury stages.

### 3.4. The Female Bias in FAP-Directed HO Is Driven by Cell-Autonomous Factors

Sex dimorphism in HO responses could represent an intrinsic difference in the skeletal progenitors, a cell-non-autonomous difference in the tissue or systemic environment, or both. To test whether male or female gonadally derived sex hormones directly or indirectly affect the HO response, 8- to 12-week-old FOP mice of the genotype *Acvr1^tnR206H/+^*;*R26^NG/+^*;Tie2-Cre were either castrated or ovariectomized, and the HO response quantified after pinch injury, which was performed 3 to 4 weeks after surgery. Although HO volumes in castrated males trended higher and showed increased variability compared to controls, neither castration nor ovariectomy resulted in a statistically significant change in HO volumes in response to pinch injury (Supplementary Figure S3). These data suggest that gonadally derived sex hormones do not play a determinative role in the observed sex-specific differences in the HO response to injury.

**Figure 4:**
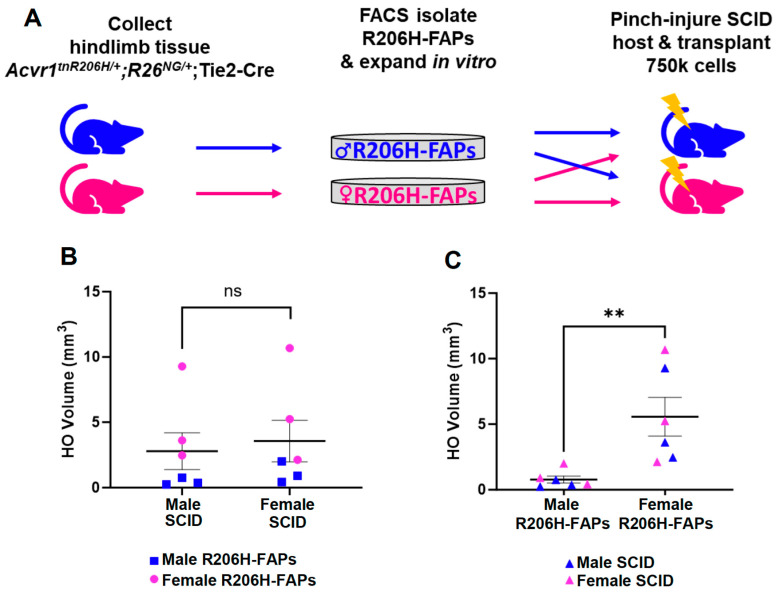
The sex difference in osteogenic response of transplanted R206H-FAPs is cell-autonomous. (**A**) Experimental workflow for the reciprocal transplantation of male and female R206H-FAPs into the GA muscle of male and female SCID hosts. (**B**) Quantification of HO volumes as a function of the sex of the SCID hosts. (**C**) Quantification of HO volumes as a function of the sex of the transplanted cells; n = 3 for males and females. A two-tailed unpaired *t*-test was used for all statistical analysis. ** *p* < 0.01. ns; not significant. Mean ± SEM.

We next designed a reciprocal transplantation experiment to further address the relative contributions of cell-autonomous and cell-non-autonomous factors (Figure 4A). R206H-FAPs were isolated from *Acvr1^tnR206H/+^*;*R26^NG/+^*;Tie2-Cre mice using FACS, expanded in culture, and 7.5 × 10^5^ cells were transplanted into the medial head of the GA muscle of SCID hosts immediately after pinch injury. Each male and female host received male cells in one limb and female cells in the contralateral limb to control for possible variability in responses between individual recipient mice (Figure 4A). Quantification of bone volumes at 15 days post-injury revealed no significant influence of host sex on the quantity of bone formed by either male or female R206H-FAPs (Figure 4B), a result consistent with the lack of significant effect of manipulating sex hormone levels in FOP mice (Supplementary Figure S3). In contrast, transplanted female R206H-FAPs formed more bone than male cells in both male and female hosts (Figure 4C; *p* < 0.01). As with FOP mice, female R206H-FAPs exhibited greater variability in osteogenic response than male cells (Figure 4C, *p* < 0.01). These data suggest that intrinsic differences rather than environmental influences dictate the magnitude and variability of the HO response.

### 3.5. Activin A Inhibition Attenuates HO Formation and Reduces the Stability of Nascent HO in Female FOP Mice

**Figure 5:**
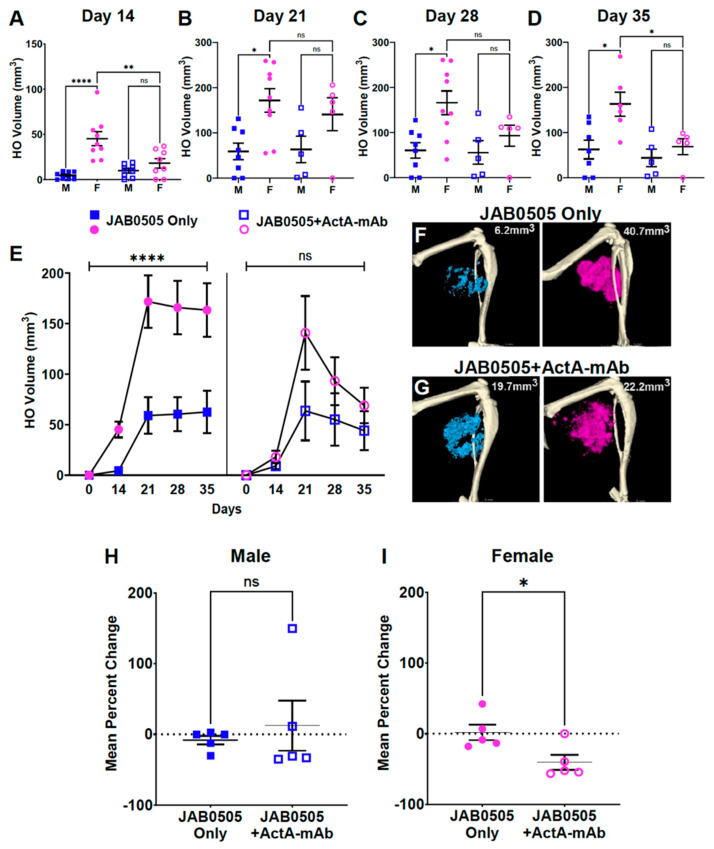
The greater HO response and partial resorption in JAB0505-treated female FOP mice is activin A-dependent. *Acvr1^tnR206H/+^;*Tie2-Cre mice were treated with JAB0505 alone or both JAB0505 and an anti-activin A neutralizing antibody (ActA-mAb), and HO volumes quantified after pinch-injury of the GA muscle. (**A**–**D**) Quantification of HO volumes of males (blue) and females (pink) at days 14, 21, 28, and 35 after muscle injury. Male and female group sizes for days 14, 21, 28, and 35, respectively, were as follows: JAB0505-treated males: n = 8, 8, 8, and 7; JAB0505-treated females: n = 9, 9, 9, and 6; JAB0505 + ActA-mAb-treated males: n = 8, 5, 5, and 5; JAB0505 + ActA-mAb-treated females: 8, 5, 5, and 5. M, male; F, female. (**E**) Line plot representation of data from (**A**–**D**) to show changes in HO formation and resorption over time. (**F**,**G**) Representative µCT images taken on day 14 post-injury display the differences in size and mineralization of HO between male (left, blue) and female (right, pink) mice treated with JAB0505 (top) or JAB0505 + ActA-mAb (bottom). (**H**,**I**) Inhibition of activin A in JAB0505-treated female mice results in partial bone resorption. The mean percentage change in HO volumes between days 21 and 35 post-injury in male (**H**) and female (**I**) mice is shown. Statistical significance was assessed using one-way (**A**–**D**) or two-way (**E**) ANOVA with multiple comparisons testing or a two-tailed unpaired *t*-test (**H,I**). * *p* < 0.05; ** *p* < 0.01; **** *p* < 0.0001. ns; not significant. Mean ± SEM.

Development of HO in FOP mice is dependent on activin A, as demonstrated by the ability of systemically delivered neutralizing monoclonal antibodies against activin A to block both injury-induced and spontaneous HO [22,30,48]. We and others have previously shown that anti-ACVR1 mAbs, while blocking ligand-dependent osteogenic signaling, are agonists of ACVR1(R206H), effectively replacing the essential function of activin A for HO formation in FOP mice [39,49]. We leveraged the agonistic activity of the anti-ACVR1 mAb, JAB0505 [39], to test whether activin A inhibition differentially affects injury-induced HO formation in male and female FOP mice. *Acvr1^tnR206H/+^*;*R26^NG/+^;*Tie2-Cre mice were treated with either 10 mg/kg JAB0505 alone or with both 10 mg/kg JAB0505 and 10 mg/kg ActA-mAb, and HO volumes were quantified by using μCT on the day of injury, and weekly from days 14 to 35 post-injury. Days 28 and 35 were included in the analysis because HO growth continues over a protracted time frame in JAB0505-treated FOP mice [39]. As expected, JAB0505 exacerbated HO in both males and females at all post-injury time points (Figure 5A–E) [39]. Female mice treated with JAB0505 alone displayed dramatically larger HO volumes than males at all time points, with the differential response being greatest at day 14 post-injury (Figure 5A–F). Notably, the inhibition of activin A in mice treated with JAB0505 differentially reduced HO in females such that no statistically significant differences in HO volumes were observed between males and females at any post-injury time point, although HO in females trended higher at all time points after day 14 (Figure 5A–E,G). 

A delayed dosing study with an ActA-mAb in FOP mice suggested that activin A is continuously required to maintain peak volumes of HO [48]. In the present study, while bone resorption was not observed in FOP mice treated with JAB0505 alone, female mice treated with JAB0505 and ActA-mAb exhibited a significant decrease in bone volume by the day 35 endpoint relative to peak volume at day 21 post-injury (Figure 5E,H,I). Collectively, these data support a role for activin A in maintaining peak volumes of nascent HO and indicate that female mice are more susceptible than males to partial HO resorption when activin A is inhibited.

In untreated FOP mice, heterotopic skeletal tissue at 14 days post-injury is comprised almost entirely of mineralized bone, sometimes with small, localized regions of cartilage remaining at the periphery of bony lesions [30]. In contrast, in JAB0505-treated FOP mice, the period of cartilage development and maturation was greatly protracted, and day 14 lesions were much larger and comprised primarily of cartilage [39] (Supplementary Figure S4). As a direct or indirect consequence, the primary period of osteogenic differentiation and mineralization was extended to day 21 post-injury [39] (Figure 5E). At day 14 post-injury, under conditions of activin A inhibition, HO volumes were essentially identical in JAB0505-treated male and female FOP mice (Figure 5A,E). Interestingly, however, the rate of the production of mineralized bone between days 14 and 21, which represented the phase of most rapid bone accumulation, was not altered by the inhibition of activin A in either males or females (compare slopes of lines in Figure 5E). These observations suggest that in female FOP mice treated with JAB0505, activin A is primarily required for the early expansion of skeletal progenitors and the production and growth of cartilage anlagen, which are ultimately replaced by bone. A role for activin A in the early population expansion of R206H-FAPs is also supported by previous cell transplantation studies [31]. Although a requirement for activin A throughout the process of heterotopic bone formation and maturation has been documented [48], the present study suggests that if ACVR1(R206H) can signal via other means, the female requirement for activin A in HO growth is restricted largely to stages prior to the main phases of osteogenic differentiation and maturation.

### 3.6. Biological Sex Impacts on BMP-Induced HO and the Effect of Activin A Inhibition

**Figure 6:**
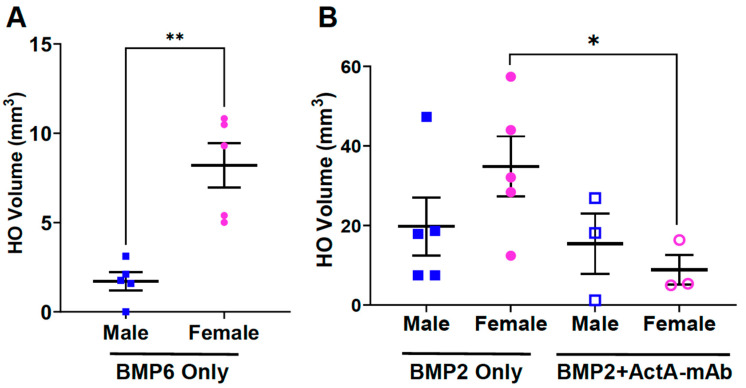
Biological sex impacts BMP-induced HO and the effect of activin A inhibition. (**A**) Quantification of HO volumes 14 days after implantation of rhBMP6 into the GA muscle of wild-type mice; n = 5 for males and females. (**B**) Quantification of HO volumes of male and female *Acvr1^tnR206H/+^*;*R26^NG/+^*;Tie2-Cre mice 14 days after implantation of rhBMP2 alone (left; n = 5 for males and females) or rhBMP2 + ActA-mAb (right; n = 3 for males and females). Statistical analyses utilized a two-tailed unpaired *t*-test. * *p* < 0.05; ** *p* < 0.01. Mean ± SEM.

To determine whether the female bias in the HO response is specific to FOP mice, the GA muscle of wild-type mice was injected with 2.5 μg rhBMP6 in a 1% alum adjuvant carrier and HO was quantified using μCT at day 14 post-injection. Female wild-type mice formed significantly more BMP6-induced HO than male mice (Figure 6A), showing that the observed female-dominant sex difference in HO volumes is not restricted to FOP mice. We note that sex differences have been reported in non-FOP models of HO and in patients who experience non-FOP HO induced by various insults and disease states [6,7,8,9,10,11,12,13,14,15,16,17,18,19]. In these contexts, however, the sex difference is typically male-dominant. Further, other studies have reported indistinguishable HO responses between males and females following implantation of BMP6 or BMP2, providing a rationale for the use of only female mice in those studies [50,51]. Whether the disparate results between models represent technical differences in experimental design or analysis or other biological variables (e.g., mouse strain, qualitative or quantitative differences in the immune response based on the method of BMP delivery) requires further investigation. 

We also tested the effect of BMP stimulation in FOP mice. BMP6 signals predominantly through ACVR1 [52,53] and competes with activin A for the activation of ACVR1(R206H)-containing complexes [22,23]. For this reason, these experiments utilized rhBMP2, which primarily signals through the type 1 receptors ALK3 and ALK6 [54]. The BMP2-induced HO response trended higher in females, but the difference between sexes did not reach statistical significance (Figure 6B). Importantly, simultaneous treatment with ActA-mAb significantly reduced the HO response in females but not males (Figure 6B). Consistent with this result, the neutralization of activin A in wild-type female mice was recently shown to attenuate HO formation induced by BMP2 and BMP6 [50,51]. Collectively, these data show that the female bias in HO formation and dependency on activin A extends to BMP-induced HO and is not restricted to JAB0505-treated mice.

## 4. Discussion

The present study demonstrated that female FOP mice exhibit a more robust and variable HO response following muscle injury than male mice. Most in vivo and cell culture models of BMP-induced osteogenesis in which sex as a variable was critically examined reported a greater degree of HO and osteogenic differentiation by male mice and cells [16,17]. However, a female bias is not restricted to FOP mouse models, as greater HO volumes were observed in female mice following intramuscular injection of BMP2 [19] and BMP6 (present study). Although the extent to which the results of the present study can be generalized to other models of FOP requires further investigation, we emphasize that the female bias in HO formation was not restricted to a narrow set of experimental conditions. Thus, increased HO in females was observed both when FAPs were targeted for *Acvr1^R206H^* expression with the Tie2-Cre driver, as well as when *Acvr1^R206H^* expression was induced much more broadly using the tamoxifen-inducible and ubiquitously expressed CAG-Cre^ERT2^ driver. Additionally, a sexually dimorphic HO response was observed for both injury-induced and spontaneous HO, using two injury modalities, when two independently derived *Acvr1^R206H^* FOP models were employed in two different mouse strains and in both young and 1-year-old adults. Taken together, these findings highlight the importance of considering sex as a variable in basic and pre-clinical studies of FOP in mouse models. 

Questionnaires have been used extensively to study the natural history of HO and other FOP disease manifestations. One study revealed that female individuals with FOP experience a far greater prevalence of neuropathic pain and other sensory abnormalities than males [55]. Analysis of patient-reported phenotypic data derived from the International FOP Association Global Registry [24] either did not find or did not address sex differences in age of symptom onset, age at diagnosis, or the number of anatomical sites functionally affected by HO [25,56]. While the prevalence of FOP is not believed to differ between males and females, an association with sex cannot be formally excluded without examining additional populations [26]. The total body burden of HO and the number of anatomical sites affected were quantified in the imaging component of a comprehensive cross-sectional natural history study of males and females with FOP [57], although the presence or absence of an association between these metrics and sex was not noted. Taken together, a relationship between sex and HO prevalence or severity cannot be formally excluded at present, although available evidence argues against a strong association. Importantly, however, even if additional studies do not support a sex bias, the sex of FOP mice should be considered a critical variable in the design, statistical powering, and reporting of basic and pre-clinical studies, given that these models are indispensable for understanding FOP pathogenesis and for evaluating the therapeutic efficacy of candidate drugs and gene therapy approaches [22,30,31,33,39,44,49,58,59,60,61,62].

Our previous work with *Acvr1^tnR206H/+^*;Tie2-Cre FOP mice identified FAPs as a major contributor to HO [30]. scRNA-seq has shown that *Tie2*+ FAPs represent a subfraction of all *Pdgfra*+ FAPs [63], and it remains unknown whether and to what extent other FAP subpopulations or additional, distinct cell types in muscle and associated connective tissues contribute to HO in FOP mice [64]. In this context, we note that greater HO volumes were observed when recombination of the disease-causing *Acvr1* allele was driven by CAG-Cre^ERT2^, which presumably activates *Acvr1^R206H^* expression in most or all cell types. This Cre-specific difference in the magnitude of the HO response may implicate cell types in addition to *Tie2*+ FAPs. Alternatively, differences in recombination efficiencies of the conditional *Acvr1^R206H^* alleles driven by Tie2-Cre and CAG-Cre^ERT2^ could explain the more robust HO response with the latter Cre driver. Given these uncertainties, the present results only formally demonstrate that *Tie2*+ FAPs exhibit female-biased sex-specific differences in HO, and further work will be necessary to determine whether *Tie2*-negative FAPs and other potential skeletal progenitors contribute to the more robust HO response in females. 

Cultured male muscle-derived stem cells (MDSCs) distinct from FAPs (see ref. [65]) exhibit greater BMP4-induced osteogenic activity than female MDSCs [18]. This difference was attributed to a greater abundance of osteoprogenitors among the MDSC fraction in male muscle tissue [18]. Here, the representation of R206H-FAPs in muscle tissue of *Acvr1^tnR206H/+^*;Tie2-Cre FOP mice was a function of the number of total FAPs, the fraction of all FAPs that express Tie2-Cre, and the efficiency of recombination of the *Acvr1^tnR206H^* allele among Tie2-Cre-expressing cells. As no significant difference between sexes was observed in the absolute number or percentage of total FAPs or R206H-FAPs in uninjured muscle, sex dimorphism in the development of HO cannot be explained by differences in R206H-FAP representation. The female bias in bone formation was also observed when equal numbers of male and female R206H-FAPs were transplanted into SCID mice, which is consistent with this conclusion. 

In both wild-type and FOP mice, muscle injury stimulates FAP proliferation, which peaks between 3 and 5 days post-injury [27,66]. Stanley et al. showed that compared to wild-type mice, FAPs from FOP mice exhibit a similar proliferation rate but reduced apoptosis after muscle injury [66]. Although the FOP allele used in that study did not allow unrecombined and R206H-FAPs to be distinguished, these data suggest that the expression of *Acvr1^R206H^* interferes with the TNFα-dependent apoptosis of FAPs (see ref. [67]), thereby leading to their dysregulated expansion. In the present study, greater HO at the endpoint in females cannot be explained by the differential expansion of R206H-FAPs at the pre-skeletal post-injury stages as the total number of R206H-FAPs and their relative representation were similar in males and females at 5 days post-injury (approximately 1 day before the appearance of histologically identifiable cartilage in this model). Interestingly, while treatment of FOP mice with the anti-ACVR1 antibody JAB0505 greatly exacerbated HO in both males and females [39] (present study), the number of R206H-FAPs 5 days after injury was similar in antibody-treated and untreated FOP mice [39]. However, mice treated with JAB0505 exhibited a significantly greater number of R206H-FAPs than untreated mice by 10 days post-injury [39], and we proposed that exacerbated HO in JAB0505-treated mice may result from the non-mutually exclusive mechanisms of a prolonged period of proliferation or reduced apoptosis, a sustained period of lineage commitment to skeletal fates, or prolongation of their recruitment to growing cartilage or bone lesional tissues [39]. We speculate that one or more of these mechanisms explain, at least in part, male–female differences in HO formation. 

While sex hormones influence many aspects of skeletal biology [68], neither castration nor ovariectomy had a statistically significant effect on HO volumes at the endpoint, arguing against a determinative role for gonadally derived sex hormones. Reciprocal transplantation studies were undertaken to determine more generally whether sex differences in HO formation were due to cell-non-autonomous factors or to intrinsic differences between male and female R206H-FAPs. These studies showed that the degree of osteogenic differentiation was associated with the sex of the transplanted cells and not the sex of SCID hosts, indicating that the sex bias in HO production does not require ongoing sex-specific influences from the local or systemic environment. The maintenance of sex-specific differences in cell culture models of stem cell-mediated osteogenic differentiation [17,18] and in cell transplantation studies of skeletal muscle regeneration [69] are additional examples of the importance of cell-intrinsic factors, the nature of which remains to be determined. Gene expression profiling of R206H-FAPs may be a productive approach to identify gene products that promote or limit HO in female and male FOP mice, respectively.

Although the present studies do not formally exclude a role for sex-specific cell-non-autonomous factors operative in FOP mice, the cell transplantation results preclude an ongoing requirement for such factors during HO lesion formation. Further, cell-non-autonomous factors would need to impart cellular properties that are maintained both during cell expansion in culture and following transplantation into hosts of the opposite sex. Maintenance of the female bias following transplantation of FACS-enriched R206H-FAPs also rules out the possibility that sex differences in HO formation in FOP mice require ongoing interactions with non-FAP, *Acvr1^R206H^*-expressing cells in the environment, which include hematopoietic and endothelial cells when Tie2-Cre is used [32,34,43] and virtually all cell types when recombination is driven by CAG-Cre^ERT2^ [38]. 

As sexual dimorphism in the expression and functional effects of activin A has been documented [70,71], we sought to determine whether activin A contributed to the observed sex differences in HO development. Mice injected with an activin A-neutralizing antibody were also treated with JAB0505, which functions as an ACVR1(R206H) agonist and activates osteogenic signaling mediated by the downstream effectors SMAD1/5/8, thereby circumventing the absolute requirement for activin A [39]. Importantly, neutralizing activin A in JAB0505-treated mice significantly reduced the HO response and increased HO resorption only in females and, consequently, eliminated significant differences in HO volumes between sexes at all time points tested. JAB0505 functions as a competitive inhibitor of ligands that bind ACVR1 and ACVR1(R206H) [39] and likely reduced the occupancy of ACVR1(R206H)-containing complexes by activin A in FOP mice. Although not dispositive, these observations suggest that the sex-specific function of activin A is not mediated by ACVR1(R206H) alone. We also note that activin A forms a non-signaling complex with ACVR1 [72], so binding of activin A to complexes containing ACVR1/ACVR1 homodimers in heterozygous FOP cells (see ref. [33]) cannot explain activin A’s action. Taken together, these observations indicate that the differential response in HO formation and stability between males and females may be mediated, at least in part, by canonical activin A signaling via the activation of SMAD2/3 through its preferred type 1 and type 2 receptors (see ref. [53]). Notably, the inhibition of activin A [50], as well as TGFβ ligands [73], which also signal through SMAD2/3, significantly attenuated BMP-induced HO. While possible sex differences were not explored, these studies demonstrated that the modulation of SMAD2/3 activity plays an important regulatory role in the formation and progression of HO. Activin A regulates myriad physiological and cellular processes [74], and further investigation is required to determine how activin A signaling affects the biology of skeletal progenitors or cells in the environment, ultimately leading to sex-specific differences in HO development and stability.

## Data Availability

No publicly archived datasets were analyzed or generated during the study. All files and images of µCT data and raw flow cytometry data are available upon request to the corresponding author.

## Supplementary Materials

The following supporting information can be downloaded at: https://www.mdpi.com/article/10.3390/biom14020177/s1, (Figures S1–S4) Figure S1. HO volumes are associated with sex but not weight of pinch-injured *Acvr1^tnR206H/+^*;*R26^NG/+^*;Tie2-Cre mice. Figure S2. Time course of average HO volumes after pinch-injury of *Acvr1^tnR206H/+^*;*R26^NG/+^*;Tie2-Cre mice. Figure S3. Castration and ovariectomy did not significantly change HO volumes following pinch-injury of the GA muscle of *Acvr1^tnR206H/+^;*Tie2-Cre mice. Figure S4. FOP mice treated with JAB0505 exhibit a protracted period of chondrogenic differentiation.
