# Supplementary material for: Sex as a Critical Variable in Basic and Pre-Clinical Studies of Fibrodysplasia Ossificans Progressiva
Source: Biomolecules. 2024 Feb 1;14(2):177. doi: 10.3390/biom14020177 (PMC10886767; doi:10.3390/biom14020177)
Supplement: Supplementary file 1 [file biomolecules-14-00177-s001.zip › biomolecules-2814680-supplementary.pdf]

Supplementary Materials (Figures S1-S4)

Figure S1. HO volumes are associated with sex but not weight of pinch-injured *Acvr1*$^{tnR206H/+}$; *R26*$^{NG/+}$;Tie2-Cre mice.

Figure S2. Time course of average HO volumes after pinch-injury of *Acvr1*$^{tnR206H/+}$;*R26*$^{NG/+}$;Tie2-Cre mice.

Figure S3. Castration and ovariectomy did not significantly change HO volumes following pinch-injury of the GA muscle of *Acvr1*$^{tnR206H/+}$;Tie2-Cre mice.

Figure S4. FOP mice treated with JAB0505 exhibit a protracted period of chondrogenic differentiation.

Supplementary Figure S1

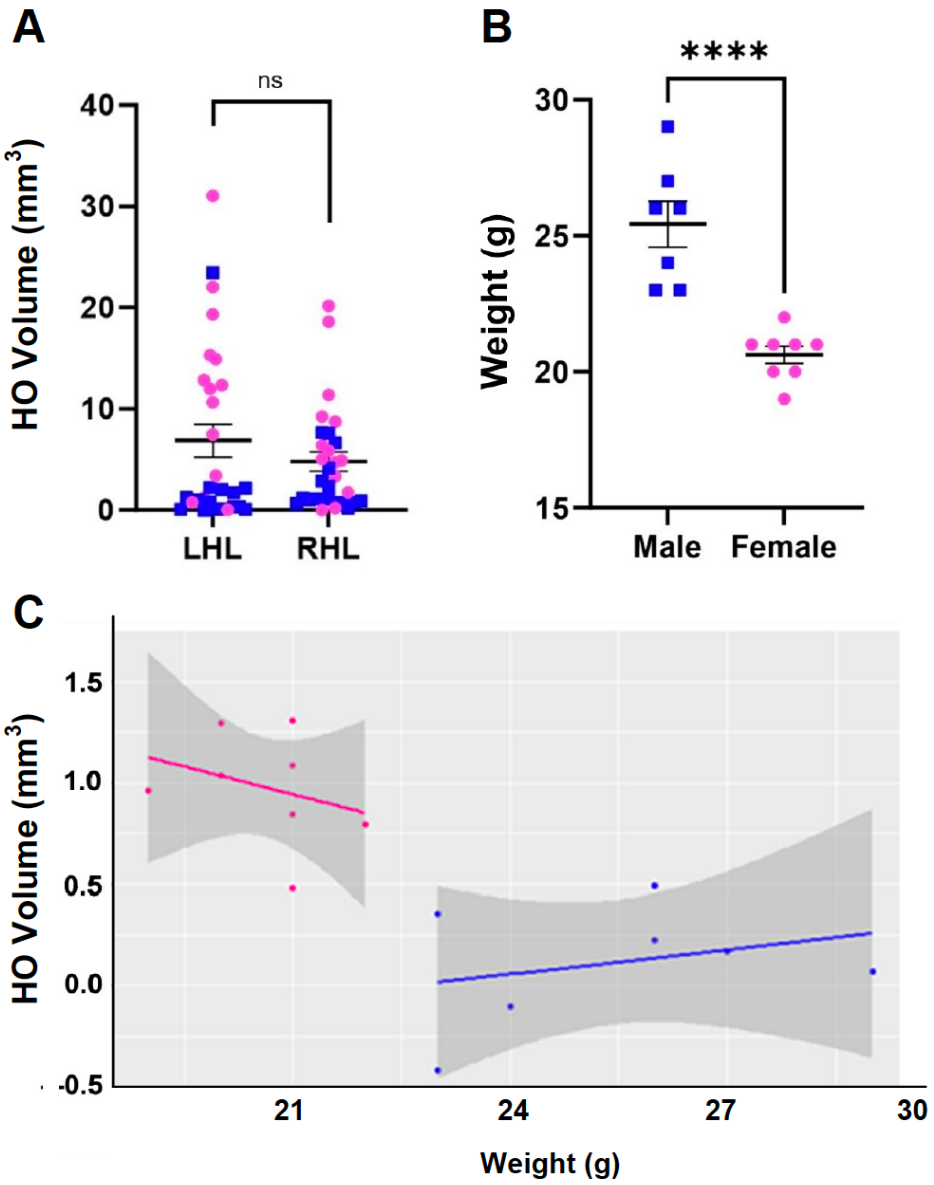

Figure S1. HO volumes are associated with sex but not weight of pinch-injured *Acvr1$^{tnR206H/+}$*; *R26$^{NG/+}$*;Tie2-Cre mice. (A) HO volumes are similar after pinch-injury of the left and right GA muscle of *Acvr1$^{tnR206H/+}$*;*R26$^{NG/+}$*;Tie2-Cre mice. LHL, left hindlimb; RHL, right hindlimb. Blue squares, males. Pink circles, females. For all experiments in which the Tie2-Cre driver was used, the calculated HO volume for each mouse represents the mean of the left and right hindlimbs. **(B)** Weight (in grams) of male and female FOP mice prior to injury. **(C)** Linear model of average HO volume with respect to sex and weight. HO volumes represented are log transformed. Slopes derived from linear model: HO = 1.57 + (0.02*Weight) – (0.95*Sex). A two-tailed unpaired t-test was used for all statistical analysis except for (C), in which linear regression analysis was performed. ** $p < 0.01$; **** $p < 0.0001$. ns; not significant. Mean ± SEM.

Supplementary Figure S2

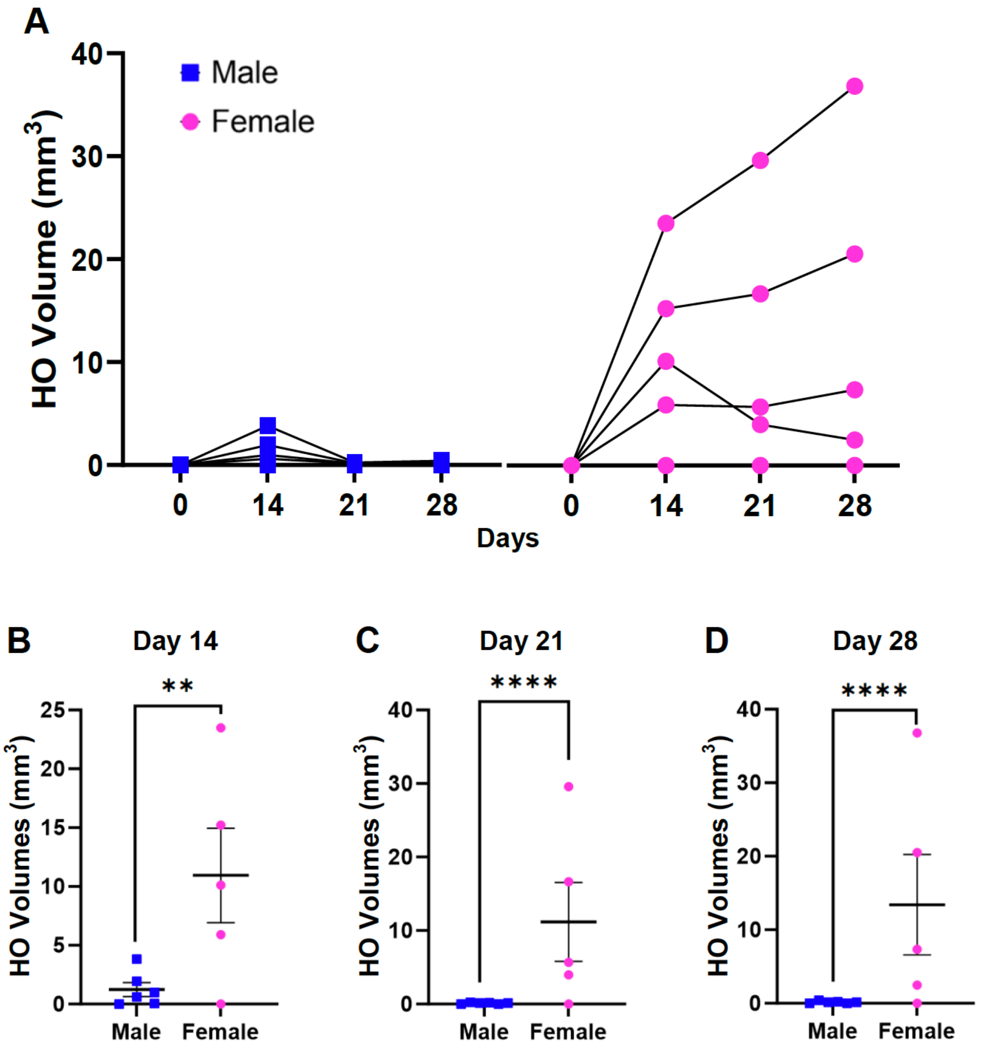

Figure S2. Time course of average HO volumes after pinch-injury of *Acvr1*$^{tnR206H/+}$;*R26*$^{NG/+}$;Tie2-Cre mice. (A) Data from Figure 1G is represented on a per mouse basis, where each line represents an individual mouse and each data point is the average of left and right hindlimb HO volumes for each mouse and time point. **(B-D)** Average HO volumes for males and females for each post-injury time point. Males, blue squares; Females, pink circles. For (B-D), an unpaired t-test was used for statistical analysis. ** $p < 0.01$; **** $p < 0.0001$. Mean ± SEM.

Supplementary Figure S3

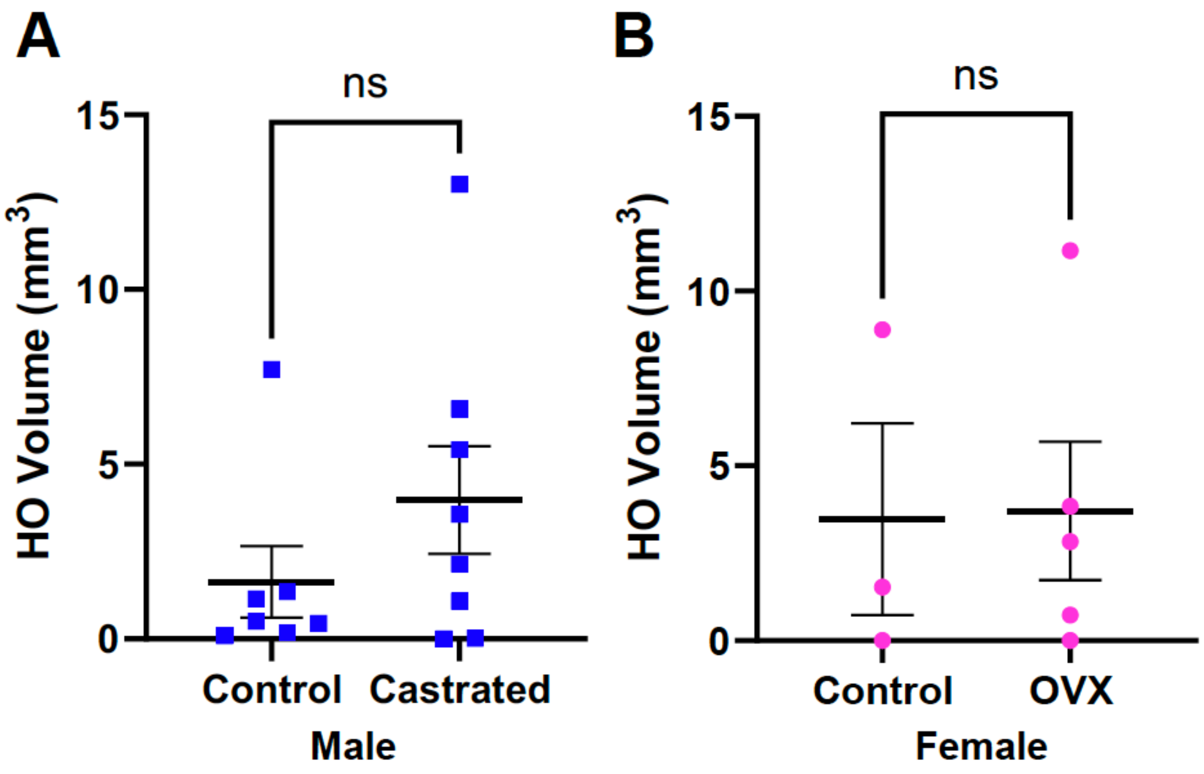

Figure S3. Castration and ovariectomy did not significantly change HO volumes following pinch-injury of the GA muscle of *Acvr1*$^{tnR206H/+}$;Tie2-Cre mice. (A) Quantification of HO volumes 21 days after injury of castrated and control males. n = 7 for control males; n = 8 for castrated males. p = 0.243. **(B)** Quantification of HO volumes 21 days after injury of ovariectomized (OVX) and control females. n = 3 for control females; n = 5 for ovariectomized females. p = 0.95. A two-tailed unpaired t-test was used for statistical analysis. Mean ± SEM.

Supplementary Figure S4

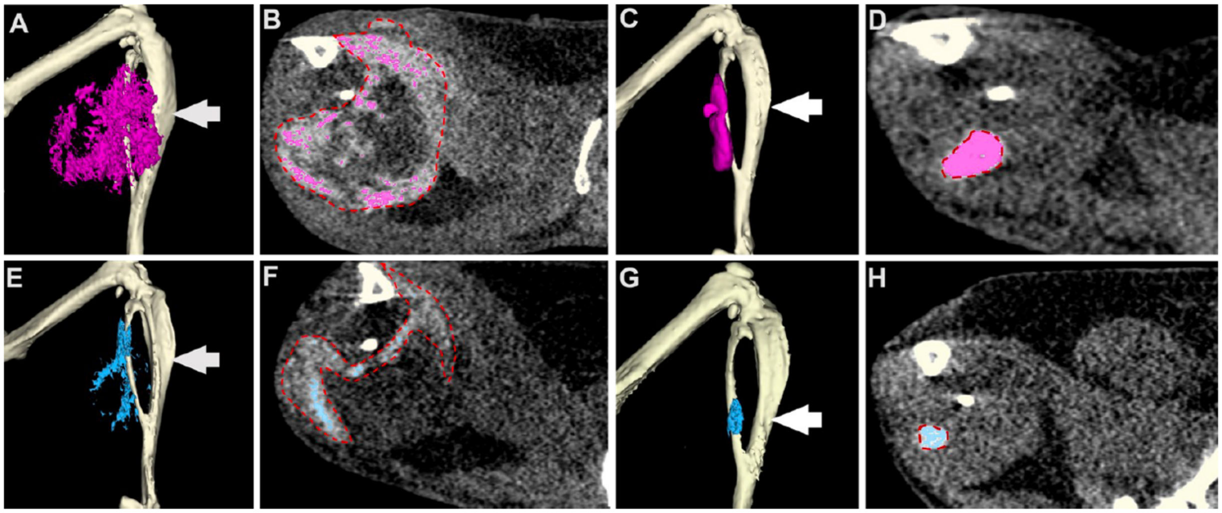

Figure S4. FOP mice treated with JAB0505 exhibit a protracted period of chondrogenic differentiation. (A, B, E, F) 3D reconstructed (A, E) and paired single transverse slice (B, D) µCT images of the distal hindlimb of female (A, B) and male (E, F) *Acvr1*$^{tnR206H/+}$;Tie2-Cre mice treated with JAB0505 and imaged 14 days after pinch-injury of the GA muscle. **(C, D, G, H)** Similar imaging of untreated *Acvr1*$^{tnR206H/+}$;Tie2-Cre mice 14 days post-injury. White arrows in (A, C, E, and G) indicate the position of the lesion represented in the corresponding cross sections. Mineralized HO is pseudocolored pink and blue for females and males, respectively. Essentially all skeletal tissue is represented by mineralized bone in untreated mice at day 14 post-injury (D and H). Radio-opaque lesional tissue below the threshold set for quantification of mineralized bone (presumptive cartilage) is extensive at day 14 in JAB0505-treated mice (B, F), giving HO a speckled, discontinuous, appearance in 3D reconstructions (A, E). The approximate boundary of lesional tissue is demarcated by red dashed lines in (B, F, D and H). The intensely radio-opaque structures (unlabeled) in (B, D, F, and H) are the tibia and fibula.
